# Vaccination Coverage Among Adults, Excluding Influenza Vaccination — United States, 2013

**Published:** 2015-02-06

**Authors:** Walter W. Williams, Peng-Jun Lu, Alissa O’Halloran, Carolyn B. Bridges, David K. Kim, Tamara Pilishvili, Craig M. Hales, Lauri E. Markowitz

**Affiliations:** 1 Immunization Services Division, National Center for Immunization and Respiratory Diseases, CDC; 2 Division of Bacterial Diseases, National Center for Immunization and Respiratory Diseases, CDC; 3 Division of Viral Diseases, National Center for Immunization and Respiratory Diseases, CDC; 4 Division of Sexually Transmitted Disease Prevention, National Center for HIV/AIDS, Viral Hepatitis, STD, and TB Prevention; CDC

Vaccinations are recommended throughout life to prevent vaccine-preventable diseases and their sequelae. Adult vaccination coverage, however, remains low for most routinely recommended vaccines (1) and below *Healthy People 2020* targets.* In October 2014, the Advisory Committee on Immunization Practices (ACIP) approved the adult immunization schedule for 2015 (2). With the exception of influenza vaccination, which is recommended for all adults each year, other adult vaccinations are recommended for specific populations based on a person’s age, health conditions, behavioral risk factors (e.g., injection drug use), occupation, travel, and other indications (2). To assess vaccination coverage among adults aged ≥19 years for selected vaccines, CDC analyzed data from the 2013 National Health Interview Survey (NHIS). This report highlights results of that analysis for pneumococcal, tetanus toxoid–containing (tetanus and diphtheria vaccine [Td] or tetanus and diphtheria with acellular pertussis vaccine [Tdap]), hepatitis A, hepatitis B, herpes zoster (shingles), and human papillomavirus (HPV) vaccines by selected characteristics (age, race/ethnicity,† and vaccination indication). Influenza vaccination coverage estimates for the 2013–14 influenza season have been published separately (3). Compared with 2012 (1), only modest increases occurred in Tdap vaccination among adults aged ≥19 years (a 2.9 percentage point increase to 17.2%), herpes zoster vaccination among adults aged ≥60 years (a 4.1 percentage point increase to 24.2%), and HPV vaccination among males aged 19–26 years (a 3.6 percentage point increase to 5.9%); coverage among adults in the United States for the other vaccines did not improve. Racial/ethnic disparities in coverage persisted for all six vaccines and widened for Tdap and herpes zoster vaccination. Increases in vaccination coverage are needed to reduce the occurrence of vaccine-preventable diseases among adults. Awareness of the need for vaccines for adults is low among the general population, and adult patients largely rely on health care provider recommendations for vaccination. The Community Preventive Services Task Force and the National Vaccine Advisory Committee have recommended that health care providers incorporate vaccination needs assessment, recommendation, and offer of vaccination into every clinical encounter with adult patients to improve vaccination rates and to narrow the widening racial/ethnic disparities in vaccination coverage (4,5).

The NHIS collects information about the health and health care of the noninstitutionalized U.S. civilian population using nationally representative samples. In-person interviews are conducted by the U.S. Census Bureau for CDC’s National Center for Health Statistics. Questions about receipt of vaccinations recommended for adults are asked of one randomly selected adult within each family in the household. The presence of selected high-risk conditions,§ as defined by ACIP for pneumococcal disease, was determined by responses to questions in the NHIS (2). Comprehensive information on all high-risk conditions for hepatitis B or A were not collected in the 2013 NHIS. Analyses were conducted to estimate age at first dose of HPV vaccination using data being collected in the NHIS for the first time starting in 2013. The final sample adult component response rate for the 2013 NHIS was 61.2%. Weighted data¶ were used to produce national vaccination coverage estimates. Point estimates and estimates of corresponding variances were calculated using statistical software to account for the complex sample design. Statistical significance was defined as p<0.05.

## Pneumococcal Vaccination Coverage

Reported pneumococcal vaccination coverage (23-valent pneumococcal polysaccharide vaccine [PPSV23] and 13-valent pneumococcal conjugate vaccine [PCV13]) among adults aged 19–64 years at high risk was 21.2% overall, similar to the estimate from 2012 (Table 1). Coverage among whites aged 19–64 years at high risk was higher (22.3%) compared with Hispanics (17.9%) and Asians (11.0%), but coverage was not significantly different for blacks (21.2%) and persons of other race (19.8%). Among adults aged ≥65 years, coverage was 59.7% overall, similar to the estimate for 2012. Coverage among whites aged ≥65 years (63.6%) was higher compared with blacks (48.7%), Hispanics (39.2%), and Asians (45.3%) (Table 1).

## Tetanus Vaccination Coverage

In 2013, the proportion of adults reporting having received any tetanus toxoid–containing vaccination during the past 10 years was 62.9% for adults aged 19–49 years, 64.0% for adults aged 50–64 years, and 56.4% for adults aged ≥65 years (Table 1). The proportion of adults receiving tetanus vaccination during the past 10 years across all age groups did not change compared with 2012 (1). Whites had higher coverage across all age groups compared with blacks, Hispanics, and Asians.

Among adults aged ≥19 years for whom Tdap vaccination specifically could be assessed (including adults aged ≥65 years), overall reported coverage was 17.2%, a 2.9 percentage point increase compared with 2012 (Table 1). Tdap coverage for black (12.6%), Hispanic (10.2%), and Asian (15.5%) adults aged ≥19 years was lower compared with whites (19.7%). Coverage among adults aged ≥19 years who reported living with an infant aged <1 year** was 29.4%, higher than the 16.7% coverage among adults aged ≥19 years without household contact with an infant aged <1 year.

Among 14,159 respondents who reported receiving a tetanus vaccination during 2005–2013, 51.2% reported that they were not informed of the vaccination type, and 10.6% could not recall what type of tetanus vaccination they had received (Table 2). Of the remaining 38.2% of respondents who reported they knew what type of tetanus vaccine they received, 68.3% reported receiving Tdap.

During 2005–2013, Tdap vaccination of health care personnel (HCP) aged ≥19 years was 37.3%, a 5.9 percentage point increase compared with 2012 (Table 3). White HCP had higher Tdap coverage (39.9%) compared with black HCP (32.2%) and Hispanic HCP (29.5%).

Among adults aged ≥19 years who received a tetanus vaccination and reported they knew what type of tetanus vaccine they received, HCP were more likely to report receipt of Tdap (76.9%) than were non-HCP (66.5%) (Table 2).

## Hepatitis A Vaccination Coverage

In 2013, reported hepatitis A vaccination coverage (≥2 doses) among adults was 9.0% for adults aged ≥19 years, 12.3% among adults aged 19–49 years, and 5.4% among adults aged ≥50 years, similar to the estimates for 2012 (Table 1). Among adults aged 19–49 years, coverage was higher for Asians (16.1%) than for whites (12.6%), but coverage for Hispanics (10.6%) was lower than for whites. Vaccination coverage was higher among adults aged ≥19 years who had traveled outside the United States since 1995 to a country where hepatitis A is of high or intermediate endemicity (countries other than Japan, Australia, New Zealand, Canada, and the countries of Europe) than among respondents who did not travel outside the United States or had traveled only to countries where the disease is of low endemicity (15.9% versus 5.7%, respectively). Vaccination coverage among adult travelers to countries with high endemicity was similar to the estimate for 2012 (Table 1). Overall coverage among adults aged ≥19 years with chronic liver conditions was 13.3%, similar to the 2012 estimate.

## Hepatitis B Vaccination Coverage

Reported hepatitis B vaccination coverage (≥3 doses) among adults was 25.0% for adults aged ≥19 years, 32.6% among adults aged 19–49 years, and 16.1% among adults aged ≥50 years. Overall vaccination coverage decreased compared with 2012 among adults aged ≥19 years by 2.1 percentage points (Table 1). Vaccination coverage was higher among adults aged ≥19 years who had traveled outside the United States since 1995 to a country where hepatitis B is of high or intermediate endemicity (countries other than Japan, Australia, New Zealand, Canada, and the countries of Europe) than among respondents who did not travel outside the United States or had traveled only to countries where hepatitis B is of low endemicity (33.1% versus 20.9%, respectively). Among adults aged 19–49 years, vaccination coverage was lower for blacks (30.5%) and Hispanics (23.7%) compared with whites (35.2%), but higher for Asians (39.3%). Overall coverage among adults aged ≥19 years with chronic liver conditions was 34.0%, similar to the 2012 estimate. Vaccination coverage for persons with diabetes was 26.3% for those aged 19–59 years and 13.9% for those aged ≥60 years, similar to the estimates for 2012. Overall, hepatitis B vaccination coverage among HCP was 61.7%, similar to the estimate for 2012. Hispanic HCP had lower coverage (54.0%) compared with white HCP (62.9%) (Table 3).

## Herpes Zoster Vaccination Coverage

In 2013, 24.2% of adults aged ≥60 years reported receiving herpes zoster vaccination to prevent shingles, an increase from the 20.1% reported in 2012 (Table 1). Whites aged ≥60 years had higher herpes zoster vaccination coverage (27.4%) compared with blacks (10.7%) and Hispanics (9.5%).

## HPV Vaccination Coverage

In 2013, 36.9% of women aged 19–26 years reported receipt of ≥1 dose of HPV vaccine, similar to the estimate reported for 2012 (Table 1). Coverage was 44.7% among women aged 19–21 years and 32.4% among those aged 22–26 years, similar to 2012 estimates. Among women aged 19–26 years, blacks (30.6%), Hispanics (30.3%), and Asians (19.8%) had lower coverage compared with whites (41.7%), but coverage for adults who indicated other race was similar to that of whites (43.1%). Receipt of ≥1 dose of HPV vaccine among males aged 19–26 years was 5.9%, a 3.6 percentage point increase compared with 2012. Coverage was 7.7% for males aged 19–21 years and 4.9% for those aged 22–26 years, increases of 5.3 and 2.7 percentage points, respectively, compared with 2012.

Among women aged 19–26 years, 1.0% reported receiving the first dose of HPV vaccine at age 8–10 years, 2.0% at age 11–12 years, 53.4% at age 13–17 years, 15.9% at age 18 years, and 27.6% at age 19–26 years. Among males aged 19–26 years, 9.7% reported receiving the first dose of HPV vaccine at age 8–10 years, 8.8% at age 11–12 years, 37.0% at age 13–17 years, 18.1% at age 18 years, and 26.3% at age 19–26 years. Among respondents aged 19–26 years, the difference between age reported at time of interview and age respondents indicated the first dose of HPV vaccine was received was ≥9 years for 5% of women and 23.8% of males. This would imply receipt of vaccination in 2004 or earlier, before HPV vaccine was licensed for use in 2006.

### Discussion

In 2013, estimated adult vaccination coverage in the United States for diseases other than influenza was similar to 2012, except for modest increases in Tdap vaccination for adults aged ≥19 years, herpes zoster vaccination among adults aged ≥60 years, and HPV vaccination among males aged 19–26 years, with no improvements in coverage for the other vaccines routinely recommended for adults. Vaccination coverage estimates for the three vaccines in this report that are included in *Healthy People 2020* (pneumococcal, herpes zoster, and hepatitis B [for HCP] vaccines) are below the respective target levels of 90% for persons aged ≥65 years and 60% for persons aged 18–64 years at high risk (pneumococcal vaccine [objectives IID 13.1 and IID 13.2, respectively]), 30% for persons aged ≥60 years (herpes zoster vaccine [IID 14]), and 90% (hepatitis B vaccine for HCP [IID 15.3]). In addition, racial/ethnic disparities in coverage persisted for all six vaccines in this report and widened for Tdap and herpes zoster vaccination, with higher coverage for whites compared with other groups. These data indicate little progress was made in improving adult vaccination coverage in the past year and highlight the need for continuing efforts to increase adult vaccination.

In August 2014, ACIP recommended routine use of PCV13 among adults aged ≥65 years.†† PCV13 should be administered in series with PPSV23, the vaccine currently recommended for adults aged ≥65 years. PPSV23 contains 12 serotypes in common with PCV13 and 11 additional serotypes. PCV13 vaccine has been demonstrated to reduce the risk for pneumococcal pneumonia, and both PCV13 and PPSV23 have been demonstrated to reduce the risk for invasive pneumococcal infections (6). Given the high proportion of invasive pneumococcal disease caused by serotypes unique to PPSV23, broader protection is expected to be provided through use of both PCV13 and PPSV23 in series. Adults who have already received PPSV23 and are recommended to receive PCV13 should receive PCV13 at lease 1 year after PPSV23 vaccine. The 2013 NHIS did not estimate the proportion of pneumococcal vaccinations by type (PCV13 versus PPSV23). The overall pneumococcal vaccination estimates in this report includes respondents who might have received PCV13 or PPSV23.

In 2012, ACIP updated the adult Tdap vaccination recommendation to include all adults aged ≥19 years who have not yet received a dose of Tdap, including those aged ≥65 years (6). Tdap, when indicated, should be administered regardless of interval since the last Td vaccination. Although there was a modest increase in overall Tdap vaccination of adults, coverage remained low for all age groups and among adults living with an infant aged <1 year. Health care providers should not miss an opportunity to vaccinate adults aged ≥19 years who have not received Tdap previously.

In December 2011, ACIP recommended that all previously unvaccinated adults aged 19–59 years with diabetes mellitus (type 1 and type 2) be vaccinated against hepatitis B as soon as possible after a diagnosis of diabetes is made, and that unvaccinated adults aged ≥60 years with diabetes may be vaccinated at the discretion of the treating clinician after assessing their risk and the likelihood of an adequate immune response to vaccination (6). Hepatitis B vaccination coverage in 2013 among persons with diabetes remained similar to estimates obtained before this recommendation and highlights the need to improve awareness of increased risk for contracting acute hepatitis B among persons with diabetes and to increase hepatitis B vaccination in this population.

ACIP recommends herpes zoster vaccination for adults aged ≥60 years.§§ Herpes zoster vaccination coverage increased in 2013 compared with 2012, with the 2013 estimate 6 percentage points below the *Healthy People 2020* target of 30%. Shortages of herpes zoster vaccine that might have contributed to lower coverage during the first years after licensure appear to have been resolved in 2012. The cost of herpes zoster vaccine and billing challenges might pose barriers for some patients and providers.¶¶

Although the percentage of age-eligible females who reported having received HPV vaccine increased steadily from 2009 to 2012, coverage did not increase further in 2013 and remained low. ACIP recommends routine vaccination of adolescent girls and boys at ages 11 or 12 years and catch-up vaccination for females aged 13–26 years who have not been previously vaccinated (6). ACIP recommends vaccination for males aged 13–21 years who have not been vaccinated previously or who have not completed the 3-dose series; males aged 22–26 years may be vaccinated (6). Data on age at first dose of HPV vaccination of adults was collected for the first time in 2013. The findings in this report indicate that most female and male respondents in the NHIS reported receiving the first dose of HPV vaccine at age ≥13 years (i.e., at ages 13–18 years). Only 2% of females and about 9% of males reported receiving the first dose at the target ages of 11 or 12 years. Some respondents also indicated the first HPV vaccination dose was received before HPV vaccine was licensed for use in 2006 suggesting inaccurate recall. In 2013, white women reported higher HPV coverage than black, Hispanic, or Asian women. The finding for Hispanic women is in contrast to data on HPV vaccination of adolescent girls aged 13–17 years reported in the 2013 National Immunization Survey – Teen (NIS-Teen) (7). In the 2013 NIS-Teen, among females, ≥1, ≥2, and ≥3 HPV dose coverage was higher among Hispanic compared with white adolescents. HPV vaccination coverage for ≥1 and ≥2 doses was higher for females living below poverty level compared with those living at or above the poverty level. The higher coverage in NIS-Teen among Hispanic females and those living below poverty level might be partly attributable to the continued effectiveness of the Vaccines for Children program, which provides recommended vaccines at no cost to eligible children through age 18 years (7). Although vaccination coverage among persons aged 13–17 years has increased since a licensed HPV vaccine has been available and recommended by ACIP, many adolescent and young adult females remain unvaccinated and vulnerable to develop cancers that HPV vaccines can prevent. Until HPV vaccination increases among adolescents, a high proportion of young women eligible for HPV vaccination will be expected. Results from studies of the cost-effectiveness of HPV vaccination of young women have suggested that catch-up vaccination could reduce the amount of time needed to achieve population level impacts of vaccination (8,9). Findings from initial studies of vaccination impact in settings where catch-up vaccination programs were successful in achieving high coverage rates among young women are consistent with these cost-effectiveness studies (9). Continued efforts are needed to ensure coverage among members of the primary target group for HPV vaccine, girls and boys aged 11 or 12 years, and among all racial/ethnic groups. Efforts are also needed to improve catch-up vaccination among those who have not started or completed their vaccinations.

What is already known on this topic?

During 2008–2012, National Health Interview Survey (NHIS) data indicated that coverage with routinely recommended vaccinations among U.S. adults aged ≥19 years remained low.

What is added by this report?

Based on 2013 NHIS data, compared with 2012, modest gains occurred in tetanus and diphtheria toxoid with acellular pertussis vaccine (Tdap) vaccination among adults aged ≥19 years (a 2.9 percentage point increase to 17.2%), herpes zoster vaccination among adults aged ≥60 years (a 4.1 percentage point increase to 24.2%), and human papillomavirus vaccination coverage among males aged 19–26 years (a 3.6 percentage point increase to 5.9%). Coverage for other vaccines and risk groups did not improve, and racial/ethnic disparities persisted for routinely recommended adult vaccines. Coverage for all vaccines for adults remained low.

What are the implications for public health practice?

Wider use of practices shown to improve adult vaccination is needed, including assessment of patients’ vaccination needs by health care providers and routine recommendation and offer of needed vaccines to adults, implementing reminder-recall systems, use of standing order programs for vaccination, and assessment of practice-level vaccination rates with feedback to staff members.

The findings in this report are subject to at least five limitations. First, the NHIS sample excludes persons in the military and those residing in institutions, which might result in underestimation or overestimation of vaccination coverage levels. Second, the response rate was 61.2%. A low response rate can result in nonresponse bias if respondents and nonrespondents differ in their vaccination rates. Third, the determination of vaccination status and identification of high-risk conditions in the NHIS were not validated by medical records. Fourth, self-report of vaccination might be subject to recall bias. Adult self-reported vaccination status has been shown to be sensitive for all six vaccines in this report and specific for all except tetanus vaccination (10). Finally, the Tdap estimate is subject to considerable uncertainty. Respondents who reported a tetanus vaccination but were unable to say whether Td or Tdap was used during 2005–2013 were excluded from estimations of Tdap coverage, creating a potential for bias. Sensitivity calculations were conducted to assess the magnitude of potential bias. Depending on what proportion of excluded respondents actually received Tdap, actual Tdap coverage could fall within the range of 13.0%–42.4% for adults aged 19–64 years and 8.7%–35.3% for adults aged ≥65 years. Comparisons of Tdap coverage across years within subgroups might be affected by bias resulting from excluding persons who did not report the type of tetanus vaccine they received.

Vaccination coverage levels among adults are low. Improvement in adult vaccination is needed to reduce the health consequences of vaccine-preventable diseases among adults. Successful vaccination programs combine 1) education of potential vaccine recipients and publicity to promote vaccination, 2) increased access to vaccination services in health care settings, and 3) use of practices shown to improve vaccination coverage, including reminder-recall systems, efforts to remove administrative and financial barriers to vaccination, use of standing order programs for vaccination, and assessment of practice-level vaccination rates with feedback to staff members (4). Health care provider recommendations for vaccination are associated with patients’ receipt of vaccines.*** Routine assessment of adult patient vaccination needs, recommendation, and offer of needed vaccinations for adults should be incorporated into routine clinical care of adults (4,5). The adult immunization schedule (2), updated annually, provides current recommendations for vaccinating adults and a ready resource for persons who provide health care services for adults in various settings.

## Figures and Tables

**TABLE 1:** Estimated proportion of adults aged ≥19 years who received selected vaccinations, by age group, high-risk (HR) status,* and race/ethnicity† — National Health Interview Survey, United States, 2013

Characteristics	Sample size	%	(95% CI)	Difference from 2012
**Pneumococcal vaccination, ever** §				
19–64 yrs, HR, total	8,988	21.2	(20.2–22.3)	1.2
19–64 yrs, HR, white	5,476	22.3	(21.0–23.8)	0.9
19–64 yrs, HR, black	1,532	21.2	(18.9–23.8)	1.5
19–64 yrs, HR, Hispanic	1,339	17.9	(15.4–20.7)¶	4.1**
19–64 yrs, HR, Asian	324	11.0	(7.7–15.3)¶	−2.3
19–64 yrs, HR, others	317	19.8	(15.1–25.5)	−0.4
≥65 yrs, total	7,433	59.7	(58.3–61.2)	−0.2
≥65 yrs, white	5,270	63.6	(61.9–65.4)	−0.3
≥65 yrs, black	984	48.7	(44.8–52.6)¶	2.5
≥65 yrs, Hispanic	724	39.2	(34.7–43.8)¶	−4.3
≥65 yrs, Asian	322	45.3	(38.6–52.0)¶	4.0
≥65 yrs, others	133	54.6	(43.8–65.0)	9.9
**Tetanus vaccination, past 10 yrs** ††				
19–49 yrs, total	16,845	62.9	(61.8–64.0)	−1.3
19–49 yrs, white	8,890	69.0	(67.7–70.4)	−0.7
19–49 yrs, black	2,506	54.1	(51.6–56.6)¶	−1.9
19–49 yrs, Hispanic	3,777	52.5	(50.4–54.6)¶	−1.4
19–49 yrs, Asian	1,222	52.7	(49.0–56.4)¶	−1.6
19–49 yrs, others	450	66.0	(59.7–71.8)	−5.9
50–64 yrs, total	8,366	64.0	(62.6–65.4)	0.5
50–64 yrs, white	5,394	67.3	(65.6–69.0)	−0.2
50–64 yrs, black	1,365	54.4	(51.0–57.7)¶	2.0
50–64 yrs, Hispanic	1,044	55.0	(50.8–59.1)¶	2.7
50–64 yrs, Asian	368	53.4	(47.3–59.4)¶	5.2
50–64 yrs, others	195	69.7	(60.5–77.6)	−0.1
≥65 yrs, total	7,236	56.4	(54.9–57.8)	1.2
≥65 yrs, white	5,111	59.6	(57.9–61.3)	1.9
≥65 yrs, black	964	40.3	(36.0–44.7)¶	−4.3
≥65 yrs, Hispanic	719	45.3	(40.7–50.0)¶	0.5
≥65 yrs, Asian	314	42.8	(36.3–49.5)¶	−3.0
≥65 yrs, others	128	72.4	(62.4–80.5)¶	22.2**
**Tetanus vaccination including pertussis vaccine, past 8 yrs** §§				
≥19 yrs, total	22,464	17.2	(16.5–17.9)	2.9**
≥19 yrs, white	12,992	19.7	(18.8–20.6)	3.6**
≥19 yrs, black	3,497	12.6	(11.1–14.2)¶	2.7**
≥19 yrs, Hispanic	3,972	10.2	(9.0–11.4)¶	1.5
≥19 yrs, Asian	1,466	15.5	(13.1–18.2)¶	0.8
≥19 yrs, others	537	22.4	(17.7–27.9)	0.9
≥19 yrs, living with an infant aged <1 yr	738	29.4	(25.7–33.3)	3.4
≥19 yrs, not living with an infant aged <1 yr	21,726	16.7	(16.0–17.4)	2.9**
19–64 yrs, total	17,356	18.4	(17.6–19.2)	2.8**
19–64 yrs, white	9,502	21.6	(20.6–22.6)	3.4**
19–64 yrs, black	2,747	13.6	(11.9–15.5)¶	3.2**
19–64 yrs, Hispanic	3,442	10.5	(9.2–11.8)¶	1.2
19–64 yrs, Asian	1,208	16.2	(13.6–19.1)¶	−0.1
19–64 yrs, others	457	22.8	(17.7–29.0)	0.1
19–64 yrs, living with an infant aged <1 yr	728	29.6	(25.9–33.6)	3.7
19–64 yrs, not living with an infant aged <1 yr	16,628	17.8	(17.1–18.6)	2.7**
≥65 yrs, total	5,108	11.9	(10.7–13.1)	3.9**
≥65 yrs, white	3,490	13.0	(11.6–14.5)	4.1**
≥65 yrs, black	750	6.5	(4.1–10.2)¶	0.6
≥65 yrs, Hispanic	530	7.3	(4.7–11.2)¶	4.0**
≥65 yrs, Asian	258	11.1	(6.5–18.2)	6.9**
≥65 yrs, others	80	18.3	(9.9–31.5)	—¶¶
≥65 yrs, living with an infant aged <1 yr	10	—¶¶	—¶¶	—¶¶
≥65 yrs, not living with an infant aged <1 yr	5,098	11.9	(10.7–13.2)	3.9**
**Hepatitis A vaccination (≥2 doses), ever** ***				
≥19 yrs, total	29,751	9.0	(8.5–9.5)	0.1
≥19 yrs, had traveled outside the United States to countries other than Europe, Japan, Australia, New Zealand, or Canada since 1995	9,249	15.9	(14.8–17.0)	−0.2
≥19 yrs, had not traveled outside the United States to countries other than Europe, Japan, Australia, New Zealand, or Canada since 1995	20,457	5.7	(5.3–6.1)†††	0.1
≥19 yrs, with chronic liver conditions, overall	396	13.3	(9.7–17.9)	0.2
19–49 yrs, total	14,752	12.3	(11.5–13.1)	0.1
19–49 yrs, white	7,801	12.6	(11.6–13.6)	0.4
19–49 yrs, black	2,254	11.0	(9.4–12.9)	−0.3
19–49 yrs, Hispanic	3,273	10.6	(9.3–12.1)¶	0.1
19–49 yrs, Asian	1,032	16.1	(13.3–19.5)¶	−2.6
19–49 yrs, others	392	15.2	(11.4–20.1)	−0.9
19–49 yrs, had traveled outside the United States to countries other than Europe, Japan, Australia, New Zealand, or Canada since 1995	5,360	18.8	(17.3–20.3)	−0.2
19–49 yrs, had not traveled outside the United States to countries other than Europe, Japan, Australia, New Zealand, or Canada since 1995	9,372	8.6	(7.9–9.4)†††	−0.0
19–49 yrs, with chronic liver conditions, overall	122	14.5	(8.6–23.4)	—¶¶
≥50 yrs, total	14,999	5.4	(4.9–5.9)	0.2
≥50 yrs, had traveled outside the United States to countries other than Europe, Japan, Australia, New Zealand, or Canada since 1995	3,889	11.8	(10.5–13.2)	−0.2
≥50 yrs, had not traveled outside the United States to countries other than Europe, Japan, Australia, New Zealand, or Canada since 1995	11,085	2.8	(2.4–3.2)†††	0.3
≥50 yrs, with chronic liver conditions, overall	274	12.7	(8.3–18.9)	1.5
**Hepatitis B vaccination**				
≥19 yrs, total	30,743	25.0	(24.3–25.8)	−2.1**
≥19 yrs, had traveled outside the United States to countries other than Europe, Japan, Australia, New Zealand, or Canada since 1995	9,861	33.1	(31.8–34.5)	−1.9
≥19 yrs, had not traveled outside the United States to countries other than Europe, Japan, Australia, New Zealand, or Canada since 1995	20,830	20.9	(20.1–21.7)†††	−2.3**
≥19 yrs, with chronic liver conditions, overall	417	34.0	(28.0–40.5)	4.0
19–49 yrs, total	15,582	32.6	(31.5–33.8)	−2.6**
19–49 yrs, white	8,196	35.2	(33.8–36.7)	−2.3
19–49 yrs, black	2,360	30.5	(27.9–33.2)¶	−3.7
19–49 yrs, Hispanic	3,470	23.7	(21.7–25.8)¶	−3.4**
19–49 yrs, Asian	1,143	39.3	(35.6–43.3)¶	−0.4
19–49 yrs, others	413	34.8	(28.4–41.7)	−2.6
19–49 yrs, had traveled outside the United States to countries other than Europe, Japan, Australia, New Zealand, or Canada since 1995	5,841	39.7	(37.9–41.6)	−2.5
19–49 yrs, had not traveled outside the United States to countries other than Europe, Japan, Australia, New Zealand, or Canada since 1995	9,718	28.4	(27.2–29.7)†††	−3.0**
19–49 yrs, with chronic liver conditions, overall	121	39.5	(28.2–52.0)	−0.6
≥50 yrs, total	15,161	16.1	(15.2–17.0)	−1.2
≥50 yrs, had traveled outside the United States to countries other than Europe, Japan, Australia, New Zealand, or Canada since 1995	4,020	23.3	(21.5–25.1)	−1.3
≥50 yrs, had not traveled outside the United States to countries other than Europe, Japan, Australia, New Zealand, or Canada since 1995	11,112	13.1	(12.2–14.1)†††	−1.1
≥50 yrs, with chronic liver conditions, overall	296	31.3	(24.7–38.7)	6.8
19–59 yrs, with diabetes, overall	1,288	26.3	(23.5–29.4)	−2.3
≥60 yrs, with diabetes, overall	1,948	13.9	(12.0–16.0)	−1.2
**Herpes zoster**				
≥60 yrs, total	10,160	24.2	(22.9–25.6)	4.1**
≥60 yrs, white	7,124	27.4	(25.8–29.0)	4.6**
≥60 yrs, black	1,375	10.7	(8.5–13.3)¶	1.9
≥60 yrs, Hispanic	1,029	9.5	(7.4–12.1)¶	0.8
≥60 yrs, Asian	440	22.6	(18.2–27.7)	5.7
≥60 yrs, others	192	24.5	(16.7–34.3)	4.8
**HPV vaccination among females (≥1 dose), ever** ****				
19–21 yrs, total	684	44.7	(39.9–49.6)	0.3
22–26 yrs, total	1,393	32.4	(29.0–36.0)	4.1
19–26 yrs, total	2,077	36.9	(34.0–39.9)	2.4
19–26 yrs, white	1,072	41.7	(37.6–46.0)	−0.5
19–26 yrs, black	353	30.6	(24.9–36.8)¶	1.4
19–26 yrs, Hispanic	463	30.3	(24.9–36.4)¶	11.6**
19–26 yrs, Asian	119	19.8	(12.5–29.9)¶	4.3
19–26 yrs, others	70	43.1	(26.9–60.9)	1.9
**HPV vaccination among males (≥1 dose), ever** ****				
19–26 yrs, total	1,837	5.9	(4.6–7.6)	3.6**
19–21 yrs, total	564	7.7	(5.2–11.5)	5.3**
22–26 yrs, total	1,273	4.9	(3.6–6.8)	2.7**

**Abbreviations:** CI = confidence interval; HPV = human papillomavirus.

* Adults were considered at high risk for pneumococcal disease if they had ever been told by a doctor or other health professional that they had diabetes, emphysema, chronic obstructive pulmonary disease, coronary heart disease, angina, heart attack, or other heart condition; had a diagnosis of cancer during the previous 12 months (excluding nonmelanoma skin cancer); had ever been told by a doctor or other health professional that they had lymphoma, leukemia, or blood cancer; had been told by a doctor or other health professional that they had chronic bronchitis or weak or failing kidneys during the preceding 12 months; had an asthma episode or attack during the preceding 12 months; or were current smokers. Information on high-risk status for hepatitis B or A was not collected in 2013.

† Race/ethnicity was categorized as follows: Hispanic, black, white, Asian and “other.” In this report, persons identified as Hispanic might be of any race. Persons identified as black, white, Asian, or other race are non-Hispanic. “Other” includes American Indian/Alaska Native and multiple race. The five racial/ethnic categories are mutually exclusive.

§ Respondents were asked if they had ever had a pneumonia shot.

¶ p<0.05 by t-test for comparisons, with non-Hispanic white as the reference.

** p<0.05 by t-test test for comparisons between 2013 and 2012 within each level of each characteristic.

†† Respondents were asked if they had received a tetanus shot in the past 10 years. Vaccinated respondents included adults who received tetanus-diphtheria toxoid (Td) during the past 10 years, or tetanus, diphtheria, and acellular pertussis vaccine (Tdap) during 2005–2013.

§§ Respondents who had received a tetanus shot in the past 10 years were asked if their most recent shot was given in 2005 or later. Respondents who had received a tetanus shot since 2005 were asked if they were told that their most recent tetanus shot included the pertussis or whooping cough vaccine. Among 34,227 respondents aged ≥19 years, those without a “yes” or no” classification for tetanus vaccination status within the preceding 10 years (n = 1,780 [5.2%]) or for tetanus vaccination status during 2005–2013 (n = 1,276 [3.7%]), or those who reported tetanus vaccination during 2005–2013 but were not told vaccine type by the provider (n = 7,209 [21.1%]) or did not know vaccine type (Td or Tdap) (n = 1,498 [4.4%]) were excluded, yielding a sample of 22,464 respondents aged ≥19 years for whom Tdap vaccination status could be assessed. In February 2012, the Advisory Committee on Immunization Practices recommended Tdap vaccination for all adults aged ≥19 years, including adults aged ≥65 years.

¶¶ Estimate is not reliable because of small sample size (n<30) or relative standard error (standard error/estimates) >0.3.

*** Respondents were asked if they had ever received the hepatitis A vaccine, and if yes, were asked how many shots were received.

††† p<0.05 by t-test for comparisons between persons who had traveled outside the United States to countries other than Europe, Japan, Australia, New Zealand, or Canada since 1995, and persons who had not traveled outside the United States to these areas since 1995.

§§§ Respondents were asked if they had ever received the hepatitis B vaccine, and if yes, if they had received ≥3 doses or <3 doses.

¶¶¶ Respondents were asked if they had ever received a shingles vaccine.

**** Respondents were asked if they had ever received the HPV shot or cervical cancer vaccine.

**TABLE 2:** Type of tetanus vaccine received, and proportion that were tetanus, diphtheria, acellular pertussis (Tdap) vaccine, among adults aged ≥19 years who received a tetanus vaccination, by selected characteristics — National Health Interview Survey, United States, 2013

Characteristics	Type of vaccine received among those who received a tetanus vaccination during 2005–2013									Proportion Tdap of total tetanus vaccinations during 2005–2013*		

	No. in sample	Received Tdap		Received other tetanus vaccine		Doctor did not inform the patient		Could not recall vaccine type				
				
		%	(95% CI)	%	(95% CI)	%	(95% CI)	%	(95% CI)	No. in sample	%	(95% CI)
≥19 yrs, all adults	14,159	26.1	(25.1–27.1)	12.1	(11.4–12.8)	51.2	(50.0–52.4)	10.6	(9.8–11.4)	5,451	68.3	(66.7–70.0)
≥19 yrs, HCP†	1,584	47.0	(43.8–50.2)	14.1	(11.9–16.7)	31.2	(28.2–34.4)	7.7	(6.2–9.4)	959	76.9§	(73.1–80.3)
≥19 yrs, non–HCP	12,564	23.5	(22.4–24.6)	11.8	(11.1–12.7)	53.8	(52.4–55.1)	10.9	(10.1–11.8)	4,489	66.5	(64.5–68.4)
19–64 yrs, all adults	11,542	26.9	(25.8–28.0)	12.1	(11.3–12.9)	50.6	(49.3–51.9)	10.5	(9.7–11.3)	4,582	69.0	(67.3–70.8)
19–64 yrs, HCP	1,439	47.4	(43.9–50.9)	14.1	(11.7–16.9)	31.0	(27.9–34.4)	7.5	(5.9–9.3)	882	77.1§	(73.0–80.7)
19–64 yrs, non–HCP	10,095	24.1	(23.0–25.2)	11.8	(11.0–12.7)	53.3	(51.9–54.7)	10.8	(10.0–11.8)	3,698	67.1	(65.1–69.1)
≥65 yrs, all adults	2,617	21.6	(19.5–23.9)	12.2	(10.5–14.2)	54.9	(52.6–57.3)	11.2	(9.6–13.1)	869	63.9	(59.2–68.4)
≥65 yrs, HCP	145	41.8	(33.1–51.1)	14.6	(9.0–22.8)	33.4	(24.2–44.2)	10.2	(5.6–17.7)	77	74.2	(62.3–83.3)
≥65 yrs, non–HCP	2,469	20.5	(18.3–22.9)	12.1	(10.3–14.1)	56.2	(53.8–58.6)	11.3	(9.6–13.2)	791	62.9	(57.8–67.7)

**Abbreviations:** CI = confidence interval; HCP = health care personnel.

* Calculated by dividing number of respondents who reported receiving Tdap by the sum of those who reported receiving Tdap and those who reported receiving other tetanus vaccination; respondents who reported that the doctor did not inform them of the vaccine type they received and those who could not recall the vaccine type were excluded.

† Adults were classified as HCP if they reported they currently volunteer or work in a hospital, medical clinic, doctor’s office, dentist’s office, nursing home, or some other health care facility, including part-time and unpaid work in a health care facility, as well as professional nursing care provided in the home.

§ p<0.05 by t-test for comparisons between HCP and non-HCP.

**TABLE 3:** Estimated proportion of health care personnel (HCP)* who received selected vaccinations, by age group and race/ethnicity† — National Health Interview Survey (NHIS), United States, 2013

Characteristics	Sample size	% (95% CI)	Difference from 2012
**Tetanus vaccination including pertussis vaccine, past 8 years** §			
HCP, ≥19 yrs, total	1,965	37.3 (34.6–40.1)	5.9¶
HCP, ≥19 yrs, white	1,197	39.9 (36.3–43.6)	6.9¶
HCP, ≥19 yrs, black	318	32.2 (25.9–39.2)**	9.7
HCP, ≥19 yrs, Hispanic	255	29.5 (22.7–37.4)**	4.4
HCP, ≥19 yrs, Asian	147	32.7 (24.1–42.7)	−6.7
HCP, ≥19 yrs, others	48	46.8 (28.8–65.7)	0.7
HCP, 19–64 yrs, total	1,766	37.9 (35.0–40.9)	5.3¶
HCP, 19–64 yrs, white	1,049	40.7 (36.9–44.6)	6.2¶
HCP, 19–64 yrs, black	297	33.2 (26.8–40.4)	10.3¶
HCP, 19–64 yrs, Hispanic	241	28.6 (21.7–36.7)**	3.5
HCP, 19–64 yrs, Asian	137	33.8 (24.9–44.0)	−7.6
HCP, 19–64 yrs, others	42	48.8 (29.1–68.9)	2.8
HCP, ≥65 yrs, total	199	30.7 (23.8–38.7)	13.8¶
HCP, ≥65 yrs, white	148	32.4 (24.1–41.9)	14.9¶
HCP, ≥65 yrs, black	21	—††	—††
HCP, ≥65 yrs, Hispanic	14	—††	—††
HCP, ≥65 yrs, Asian	10	—††	—††
HCP, ≥65 yrs, others	6	—††	—††
**Hepatitis B vaccination (≥3 doses), ever** ¶¶			
HCP, ≥19 yrs, total	2,606	61.7 (59.0–64.3)	−3.3
HCP, ≥19 yrs, white	1,610	62.9 (59.4–66.2)	−2.6
HCP, ≥19 yrs, black	428	58.9 (53.2–64.3)	−2.8
HCP, ≥19 yrs, Hispanic	326	54.0 (46.9–61.0)**	−6.0
HCP, ≥19 yrs, Asian	182	69.0 (60.7–76.2)	−3.3
HCP, ≥19 yrs, others	60	56.0 (39.4–71.4)	−19.8

**Abbreviation:** CI = confidence interval.

* Adults were classified as HCP if they reported they currently volunteer or work in a hospital, medical clinic, doctor’s office, dentist’s office, nursing home, or some other health-care facility, including part-time and unpaid work in a health care facility, as well as professional nursing care provided in the home.

† Race/ethnicity was categorized as follows: Hispanic, black, white, Asian and “other.” In this report, persons identified as Hispanic might be of any race. Persons identified as black, white, Asian, or other race are non-Hispanic. “Other” includes American Indian/Alaska Native and multiple race. The five racial/ethnic categories are mutually exclusive.

§ Respondents who had received a tetanus shot in the past 10 years were asked if their most recent shot was given in 2005 or later. Respondents who had received a tetanus shot since 2005 were asked if they were told that their most recent tetanus shot included the pertussis or whooping cough vaccine. Among 2,777 HCP aged ≥19 years, those without a “yes” or no” classification for tetanus vaccination status within the preceding 10 years (n = 82 [3.0%]) or for tetanus vaccination status during 2005–2013 (n = 105 [3.8%]), or those who reported tetanus vaccination during 2005–2013 but were not told vaccine type by the provider (n = 500 [18.0%]) or did not know vaccine type (Td or Tdap) (n = 125 [4.5%]) were excluded, yielding a sample of 1,965 respondents aged ≥19 years for whom Tdap vaccination status could be assessed. In February 2012, the Advisory Committee on Immunization Practices recommended Tdap vaccination for all adults aged ≥19 years, including adults aged ≥65 years.

¶ p<0.05 by t-test for comparisons between 2013 and 2012 within each level of each characteristic.

** p<0.05 by t-test for comparisons, with non-Hispanic white as the reference.

†† Estimate is not reliable because of small sample size (n<30) or relative standard error (standard error/estimates) >0.3.

¶¶ Respondents were asked if they had ever received the hepatitis B vaccine, and if yes, if they had received ≥3 doses or <3 doses.

## Footnotes

* *Healthy People 2020* objectives and targets for immunization and infectious diseases are available at https://www.healthypeople.gov/2020/topics-objectives/topic/immunization-and-infectious-diseases/objectives.

† Race/ethnicity was categorized as Hispanic, black, white, Asian, and “other.” Persons identified as Hispanic might be of any race. Persons identified as black, white, Asian, or other race are non-Hispanic. “Other” includes American Indian/Alaska Native and multiple race. The five racial/ethnic categories are mutually exclusive.

§ Adults were considered at high risk for pneumococcal disease or its complications if they had ever been told by a doctor or other health professional that they had diabetes, emphysema, chronic obstructive pulmonary disease, coronary heart disease, angina, heart attack, or other heart condition; had a diagnosis of cancer during the previous 12 months (excluding nonmelanoma skin cancer); had ever been told by a doctor or other health professional that they had lymphoma, leukemia, or blood cancer; had been told by a doctor or other health professional that they had chronic bronchitis or weak or failing kidneys during the preceding 12 months; had an asthma episode or attack during the preceding 12 months; or were current smokers.

¶ Additional information on NHIS methods is available at http://www.cdc.gov/nchs/nhis/methods.htm.

** In 2006, a single dose of Tdap was recommended for adults who have or who anticipate having close contact with an infant aged <1 year (e.g., parents, grandparents, child-care providers, and health care personnel) to reduce the risk for transmitting pertussis.

†† Additional information on use of PCV13 among adults aged ≥65 years available at http://www.cdc.gov/mmwr/preview/mmwrhtml/mm6337a4.htm?s_cid=mm6337a4_w.

§§ Additional information on herpes zoster vaccination for adults aged ≥60 years available at http://www.cdc.gov/mmwr/preview/mmwrhtml/mm6333a3.htm?s_cid=mm6333a3_w.

¶¶ Additional information on barriers to herpes zoster vaccination available at http://www.gao.gov/products/GAO-12-61 and http://www.gao.gov/assets/590/587009.pdf.

*** Additional information on provider recommendations for vaccination available at http://onlinelibrary.wiley.com/doi/10.1111/j.1532-5415.2005.00585.x/pdf.
